# The role of human ventral visual cortex in motion perception

**DOI:** 10.1093/brain/awt214

**Published:** 2013-08-26

**Authors:** Sharon Gilaie-Dotan, Ayse P. Saygin, Lauren J. Lorenzi, Ryan Egan, Geraint Rees, Marlene Behrmann

**Affiliations:** 1 Institute of Cognitive Neuroscience, University College London, London, UK; 2 Wellcome Trust Centre for Neuroimaging, University College London, London, UK; 3 Department of Cognitive Science and Neuroscience Program, University of California San Diego, San Diego, California, USA; 4 Department of Psychology, Carnegie Mellon University, Pittsburgh, PA, USA

**Keywords:** motion detection, motion coherence, visual motion, MT+/V5, form perception, brain damage

## Abstract

Visual motion perception is fundamental to many aspects of visual perception. Visual motion perception has long been associated with the dorsal (parietal) pathway and the involvement of the ventral ‘form’ (temporal) visual pathway has not been considered critical for normal motion perception. Here, we evaluated this view by examining whether circumscribed damage to ventral visual cortex impaired motion perception. The perception of motion in basic, non-form tasks (motion coherence and motion detection) and complex structure-from-motion, for a wide range of motion speeds, all centrally displayed, was assessed in five patients with a circumscribed lesion to either the right or left ventral visual pathway. Patients with a right, but not with a left, ventral visual lesion displayed widespread impairments in central motion perception even for non-form motion, for both slow and for fast speeds, and this held true independent of the integrity of areas MT/V5, V3A or parietal regions. In contrast with the traditional view in which only the dorsal visual stream is critical for motion perception, these novel findings implicate a more distributed circuit in which the integrity of the right ventral visual pathway is also necessary even for the perception of non-form motion.

## Introduction

The visual system is traditionally segregated into two main processing streams, the ventral ‘form’ visual pathway, and the dorsal ‘visuospatial’ pathway, also sometimes referred to as the ‘motion’ pathway (Ungerleider and Mishkin, 1982; Mishkin *et al.*, 1983; Van Essen and Maunsell, 1983; Van Essen, 1985; Maunsell and Newsome, 1987; Maunsell *et al.*, 1990; Goodale and Milner, 1992). This functional and anatomical segregation in retinotopic cortex (dorsal and ventral streams representing the lower and upper hemifields, respectively) effectively extends the parvocellular and magnocellular subdivisions into the visual cortex (Livingstone and Hubel, 1987, 1988; Maunsell and Newsome, 1987; Maunsell *et al.*, 1990), where the form visual pathway represents the continuation of the parvo pathway, and the dorsal ‘visuospatial’/‘motion’ pathway continues the mango pathway (Livingstone and Hubel, 1988). Although perception of visual motion is implicated in a diverse set of behaviours such as directing attention in the visual environment or segmenting a moving object from its background, it has long been considered under the purview of the dorsal visual pathway. Indeed, anatomically and functionally, motion-sensitive cortical regions including hMT+/V5 show a clear dorsal magnocellular dominance (Maunsell *et al.*, 1990), and their critical role in motion perception is well established, as damage to hMT+/V5 adversely affects motion perception (Zihl *et al.*, 1983, 1991; Zeki, 1991; Beckers and Homberg, 1992; Rizzo *et al.*, 1995; McLeod *et al.*, 1996; Marcar *et al.*, 1997; Schenk and Zihl, 1997; Vaina *et al.*, 2001; Billino *et al.*, 2009). However, there is growing recognition that these motion sensitive areas alone may not be sufficient for normal motion perception (Majaj *et al.*, 2007; Hedges *et al.*, 2011).

Several findings indicate that the ventral pathway may also be implicated in motion perception (Ungerleider and Desimone, 1986; Treue and Maunsell, 1996; Newsome, 1997; and see an overview in Chapman *et al.*, 2004; Hayward *et al.*, 2011). For example, motion-sensitive regions (e.g. MT/MST in humans) are anatomically located at the intersection of the dorsal and ventral streams in lateral temporal cortex (Ungerleider and Mishkin, 1982; Goodale and Milner, 1992), and are thus not exclusively within the dorsal pathway. Further findings, primarily from non-human primates, indicate that ventral visual cortices may play an important functional role in motion perception (Maunsell and Van Essen, 1983; Ungerleider and Desimone, 1986; Desimone and Schein, 1987; Mountcastle *et al.*, 1987; Ferrera *et al.*, 1994; De Valois *et al.*, 2000; Ferrera and Maunsell, 2005; Tolias *et al.*, 2005; Lu *et al.*, 2010), and it has also been suggested that slower motion (<3°/s) might be supported by the ventral pathway following the sustained nature of the parvocellular system, whereas faster motion is supported by the dorsal more transient magnocellular pathway (Gegenfurtner and Hawken, 1996; Thompson *et al.*, 2006; Hayward *et al.*, 2011). It is also the case that visual motion is engaged in form-associated functions (Kourtzi *et al.*, 2008). However, what remains to be determined is whether ventral cortex is critical to motion perception, as the ventral route was not affected in previous case studies which have reported motion perception deficits (Zihl *et al.*, 1983, 1991; Vaina *et al.*, 1990, 2001; Zeki, 1991; Rizzo *et al.*, 1995; McLeod *et al.*, 1996; Marcar *et al.*, 1997; Schenk and Zihl, 1997; Billino *et al.*, 2009).

To evaluate the contribution of ventral visual cortex to motion perception, we compared the visual motion perception of five human adults with a focal lesion in ventral cortex with that of age- and gender-matched controls. Lesions were determined to be in ventral cortex for each of the patients based on neuroradiological expert opinion and on anatomical landmarks of the lesion in the ventral aspects of the occipital and temporal lobes. The lesion site in the patients was either to the left or to the right ventral visual pathway, and all patients exhibited form perception deficits (Table 1), either object agnosia and/or pure alexia. Some of the patients had preserved MT/V5, and all were tested several years after lesion onset.

**Table 1:** Summary of case histories

	Left ventral lesion		Right (+ left?)	Right ventral lesion	
Patient	EL	GB	*CR*	*SM*	*EC*
Age (gender)	61(F)	70(F)	31(M)	37(M)	48(F)
Controls’ ages (number, gender)	63 ± 3.6 (4, F)	63 ± 3.6 (4, F)	31.4 ± 3.2 (11, M)	35.9 ± 3.9 (9, M)	48.2 ± 3.5 (8, F; 1, M)
Time from injury (motion perception testing)	15 years	3 years	15 years	19 years	8 years
Visual impairments	Pure alexia, mild impairment in object recognition	Pure alexia, mild impairment in object perception	Object agnosia and prosopagnosia	Object agnosia and prosopagnosia	Face and object recognition difficulties (screening)

Further details are provided in the Supplementary material.

Because motion perception is involved in a range of perceptual tasks (Newsome and Pare, 1988; Rizzo *et al.*, 1995; McLeod *et al.*, 1996; Murray *et al.*, 2003; Noguchi *et al.*, 2005; Gilaie-Dotan *et al.*, 2011), some of which engage form representations (for example, perceiving structure-from-motion) and some of which do not (merely detecting the presence of motion in a display), we examined whether different types of motion perception were affected by the neural insult (Hedges *et al.*, 2011). We assessed performance in both basic motion tasks (motion coherence or detection; Newsome and Pare, 1988; Stoner and Albright, 1992; Albright and Stoner, 1995; Rees *et al.*, 2000; Thiele and Stoner, 2003) and in more complex motion tasks (3D structure-from-motion; Vaina *et al.*, 1990; McLeod *et al.*, 1996; Grossman *et al.*, 2000; Murray *et al.*, 2003; Saygin, 2007; Jastorff and Orban, 2009; Gilaie-Dotan *et al.*, 2011). Motion speed was also manipulated to evaluate whether ventral cortex contributed differently to the perception of slow or fast motion (Hayward *et al.*, 2011; Narasimhan and Giaschi, 2012).

To explore brain–behaviour correlations and determine whether motion perception deficits, if present, might be attributable to co-occurring lesions of MT/V5, V3A and/or parietal cortex, for each individual, we delineated the lesion site based on structural imaging data. Furthermore, given that four of the five patients had a focal unilateral lesion, we had the opportunity to explore whether damage to the right and left ventral cortices affects motion perception equivalently.

We hypothesized that, if the ventral visual cortex plays a critical role in basic motion perception, then a lesion to this region should impair motion perception across all tasks. Moreover, this should hold true even if MT/V5, V3A or parietal regions were intact. Alternatively, if ventral visual cortex is only engaged when motion contributes to form perception, then a ventral visual lesion should only impair motion perception when form representations are evoked (e.g. by structure-from-motion stimuli), but not when only basic motion perception is tapped (e.g. motion coherence or motion detection). Finally, if ventral cortex contributed only to slow motion perception, then a ventral visual lesion would impair slow but not fast motion perception.

## Materials and methods

### Participants

All participants gave written informed consent to participate and the protocol was approved by the Institutional Review Board, Carnegie Mellon University and by the University College London local ethics committee. The patients and a subset of controls were tested in Pittsburgh whereas the remaining control participants were tested in London. Patients were tested either at the university or in their home.

#### Patients

Five premorbidly normal individuals, all right-handed, participated in these experiments. After a lesion sustained in adulthood (except for CR who was aged 16 years at lesion onset), all individuals reported visual perceptual problems. Table 1 summarizes the patients’ case descriptions; more detailed information is available in the Supplementary material and in previous publications (SM: Gauthier *et al.*, 1999; Marotta *et al.*, 2001; Behrmann and Kimchi, 2003; Behrmann and Williams, 2007; Nishimura *et al.*, 2010; Konen *et al.*, 2011; Behrmann and Plaut, 2013; CR: Gauthier *et al.*, 1999; Marotta *et al.*, 2001; Behrmann and Williams, 2007; Behrmann and Plaut, 2013; EL: Behrmann *et al.*, 1998; Montant and Behrmann, 2001; McKeeff and Behrmann, 2004; Mycroft *et al.*, 2009; Behrmann and Plaut, 2013; GB: Behrmann and Plaut, 2013). Aside from EL, who has an upper right visual field quadrantanopia, the other patients all have full visual fields. In spite of her field defect, EL performed normally on all motion perception tasks. All patients had normal or corrected to normal visual acuity. No reported or apparent problems in vergence or accommodation were evident in any of the patients.

#### Control participants

A group of 36 control participants participated in this study: 11 male control participants served as age-matched controls for CR [mean age ± standard deviation (SD) = 31.36 ± 3.2 years]; nine males served as controls for SM [aged 35.89 ± 3.86 (SD) years, six of whom were matched for both CR and SM]; eight females and one male (also matched for SM) served as controls for EC [aged 48.22 ± 3.53 (SD) years]. The control group for GB and EL was composed of four females (aged 58–66 years, mean 63 ± 3.56 (SD) years). For the motion coherence parametric experiment, five females [aged 35–67 years, mean 58 ± 13 (SD) years] served as controls for EL who was aged 63 when assessed. Two of these females have also completed the rest of the study as controls for GB and EL, whereas three did not participate in the other experiments of this study. Seven males [aged 37.1 ± 3.53 (SD) years], who did not participate in the other experiments of this study served as controls for SM for the motion coherence parametric experiment, who was aged 39 when completing this experiment. All control participants were right-handed, had normal or corrected-to-normal vision and no history of neurological disorders.

### Motion perception experiments

#### Motion coherence: perceptual threshold

Non-form-based motion perception was assessed by measuring motion coherence thresholds as described elsewhere (Gilaie-Dotan *et al.*, 2013).

Stimuli were circular random dot patterns (Green, 1961; Levinson and Sekuler, 1976) displayed in the centre of the screen, each random dot pattern consisting of 500 grey dots (luminance = 2.50 Cd/m^2^, width = 2.77 minArc) against a black background (luminance = 0.37 Cd/m^2^), and covering a circular area (width = 9.13° when viewed from a distance of 52 cm). A two-interval forced choice paradigm was used. On each trial, two stimuli intervals, each 333 ms in duration, were presented in succession, with an interstimulus interval of 1000 ms. One randomly chosen interval consisted of coherent motion plus noise, and the other interval consisted only of noise. The participants’ task was to decide whether the first or second viewed interval contained more coherent motion. For the signal (coherent motion) plus noise stimulus, a randomly chosen subset of the dots (i.e. the signal) was vertically displaced upwards or rightwards by 0.45° steps in 20 consecutive frames (total motion time = 333 ms; speed = 27.27°/s, corresponding to the very fast motion speed in the parametric assessment of motion coherence, see below), so that the lifetime of the signal dots was 333 ms. The remaining dots (the noise) were repositioned randomly from one frame to the next (Scase *et al.*, 1996). Coherently moving dots reaching either end of the circular display area were repositioned on the other side for the next frame. A central fixation square (width = 0.55°) was displayed throughout the experiments. No time limit was imposed on responses. Stimuli were generated using the Cogent toolbox (http://www.vislab.ucl.ac.uk/Cogent/) for MATLAB (Mathworks) and were presented at 60 Hz using a TFT-LCD display (resolution = 800 × 600, 15.4-in screen).

**Figure 1:**
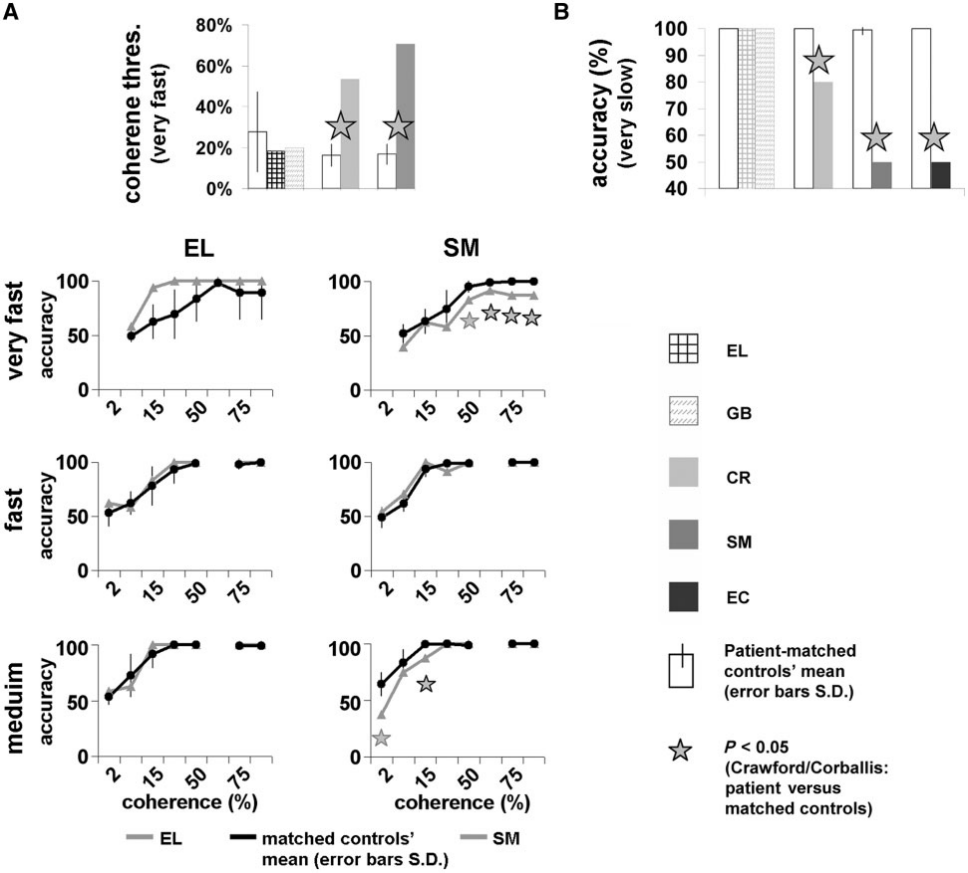
Performance on basic non-form motion perceptual tasks. Each patient’s data are plotted to the right of the matched control group. EL and GB had the same group of controls. (**A**) *Top* Motion coherence thresholds (percentage of points moving in a coherent direction needed to detect the coherent motion interval at 75% accuracy; lower thresholds correspond to better performance (see Table 2). Bar colours according to legend. *Bottom*: Motion coherence parametric performance for three speeds, for SM and EL (grey lines) and their matched controls (black lines, seven for SM, five for EL). Coherence levels (2.5, 5, 15, 25, 50, 65, 75 and 95%) on the *x*-axis, accuracy (%) on the *y*-axis. (**B**) Motion detection (very slow motion) accuracy levels (see details and EL’s performance in Table 4). Asterisks denote significant impairment of patient versus matched controls (*P* < 0.05; Crawford and Howell, 1998); Error bars = SD of control group. Apart from SM’s normal performance for intermediate speed (**A**), all right ventral patients were impaired in all motion perception tasks here, at slow and at fast speeds, whereas left ventral patients performed at ceiling.

For each observer, we determined a coherence level threshold that corresponded to 75% accuracy using the accelerated stochastic approximation method (Kesten, 1958; Treutwein, 1995). This method updated the approximation on every trial in a staircase manner, with steps becoming smaller following a change in the response accuracy; incorrect responses had a bigger effect (‘penalty’). Each run of the staircase consisted of 48 trials. Participants started with a practice session with initial coherence level of 70–90% (see ‘Results’ section for details) and a verbal explanation. Thereafter, participants performed one to two additional runs, with the first run starting with an initial coherence threshold of 50%. For the second run, the input threshold was taken as the output of the previous run. The output threshold of the last session performed was taken as the individual’s coherence threshold. We compared each patient’s coherence threshold to those of his/her corresponding control participants and determined whether performance was significantly different (Crawford and Howell, 1998; Crawford *et al.*, 2009), as presented in Table 2 and Figure 1. Additional details about motion coherence in specific directions (upwards, downwards, leftwards and rightwards) are provided in the Supplementary material.

**Table 2:** Motion coherence thresholds and statistical analysis

	Coherence threshold (%)			T_Crawford_	P_Crawford_
Patient initials	Controls (mean)	Controls (SD)	Patient		
EL	27.6	19.6	17.92	−0.404	0.755
GB	27.6	19.6	19.97	−0.31	0.803
*CR*	*16.24*	*5.31*	***53.58***	***5.73***	***0.00036***
*SM*	*16.80*	*4.97*	***70.57***	***8.84***	***0.00045***

Perceptual thresholds indicate the percentage of points that needed to move coherently so that a participant could detect correctly the coherent motion interval at 75% accuracy (see ‘Materials and methods’ section. Lower thresholds indicate better performance). Bold indicates that a patient’s coherence threshold was significantly higher (i.e. impaired) than their matching controls (*t*-test results; Crawford and Howell, 1998); right ventral lesioned patients’ data are italicized. Motion coherence thresholds of the right ventral lesioned patients (CR and SM) were significantly impaired, while those of the left hemisphere patients were normal, even when compared with younger control subjects (see Fig. 1A and Supplementary Material for more details).

#### Motion coherence: parametric assessment at different speeds

We parametrically assessed motion coherence performance for three different speeds: very fast (27.27°/s, identical to the speed in the motion coherence threshold experiment, see above, similar to Meteyard *et al.*, 2008; Gilaie-Dotan *et al.*, 2013), fast (10.8°/s, similar to speeds in previously reported paradigms: Zihl *et al.*, 1983; Rizzo *et al.*, 1995; Scase *et al.*, 1996; Noguchi *et al.*, 2005; van Boxtel and Erkelens, 2006; Pilly and Seitz, 2009; Tailby *et al.*, 2010), and medium (5.4°/s, used in previous studies: Scase *et al.*, 1996; Rees *et al.*, 2000; Giaschi *et al.*, 2003). This allowed us to assess performance for motion speeds ranging from 5.4–27.27°/s. For each of these speeds separately, we measured accuracies for varying degrees of coherently moving dots (95%, 75%, 65%, 50%, 25%, 15%, 5%, and 2%; 65% was only measured for the very fast speed to obtain an additional observation for SM at the range of 50–75%, 2% was not measured for the very fast speed due to chance level performance at 5%). Each coherence degree and specific speed was examined in a separate run consisting of 24 randomly ordered trials, 12 with upwards and 12 with downwards coherent motion. It is important to emphasize that we kept the experimental paradigm as close as possible to that of the motion coherence threshold paradigm, including the timing and duration of the trials, the size, colours and luminance of the circular display, the number, size and colour of dots in the display, and the lifetime of the dots (for the dots in the signal and the noise dots). As above, the display in each trial was a centrally displayed circle (width = 9.13° when viewed from 52 cm) of 500 grey dots (luminance = 2.50 Cd/m^2^, width = 2.77 minArc) randomly located within it against a black background (luminance = 0.37 Cd/m^2^). A proportion of the points (i.e. according to the coherence level) comprising the signal moved in a coherent fashion either upwards or downwards, and the participants’ task was to report the direction of perceived motion (upwards or downwards) using up/down key presses, or by verbal report after which the experimenter pressed the buttons accordingly.

After a verbal explanation, all participants were tested first with the medium speed, then the fast speed, followed by the very fast speed conditions. For each speed, the testing started with the highest coherence degree (95%) followed by gradually descending coherence levels. For each speed and each coherence level, we compared the patient’s performance to that of their corresponding control participants and determined whether performance was significantly different (Crawford and Howell, 1998; Crawford *et al.*, 2009), as presented in Table 3.

**Table 3:** Motion coherence parametric performance

Speed (°/s)	Coherence level		Accuracy (% correct)			T_Crawford_	P_Crawford_
		Patient initials	Controls (mean)	Controls (SD)	Patient		
27.27	95	EL	89.2	24	100	0.41	0.7
		*SM*	*100*	*0*	***87.5***	***−116.9***	***2 × 10^−^^11^***
	75	EL	89.2	24	100	0.41	0.7
		*SM*	*100*	*0*	***87.5***	***−116.9***	***2 × 10^−^^11^***
	65	EL	97.9	3	100	0.77	0.49
		*SM*	*99.2*	*1.9*	***91.7***	***−3.67***	***0.02***
	50	EL	83.3	20	100	0.75	0.49
		*SM*	*95.2*	*5.1*	***83.3***	***−2.20***	***0.07***
	25	EL	69.3	23	100	1.22	0.30
		*SM*	*74.5*	*17.7*	*58.3*	−*0.85*	*0.42*
	15	EL	62.5	16	93.8	1.78	0.17
		*SM*	*63.4*	*11.2*	*62.5*	*−0.076*	*0.942*
	5	EL	49.5	5	58.3	1.45	0.24
		*SM*	*52.1*	*8.6*	*39.6*	*−1.36*	*0.22*
10.8	95	EL	100	0	100	0	1
		*SM*	*100*	*0*	*100*	*0*	*1*
	75	EL	97.9	4	100	0.44	0.68
		*SM*	*100*	*0*	*100*	*0*	*1*
	50	EL	99.2	2	100	0.41	0.7
		*SM*	*99.2*	*1.9*	*100*	*0.41*	*0.7*
	25	EL	93.3	13	100	0.47	0.65
		*SM*	*99.2*	*1.9*	***91.7***	**−*3.67***	***0.021***
	15	EL	78.3	18	83.3	0.25	0.81
		*SM*	*94*	*7.3*	*100*	*0.75*	*0.49*
	5	EL	62.5	11	58.3	−0.35	0.73
		*SM*	*61.7*	*6.9*	*70.1*	*1.22*	*0.29*
	2	EL	53.3	12	62.5	0.67	0.53
		*SM*	*49.2*	*9.5*	*54.2*	*0.48*	*0.66*
5.4	95	EL	99.2	2	100	0.41	0.7
		*SM*	*100*	*0*	*100*	*0*	*1*
	75	EL	99.2	2	100	0.41	0.7
		*SM*	*100*	*0*	*100*	*0*	*1*
	50	EL	100	0	100	0	1
		*SM*	*98.8*	*2*	*100*	*0.54*	*0.6*
	25	EL	100	0	100	0	1
		*SM*	100	0	100	0	1
	15	EL	91.7	12	100	0.63	0.56
		*SM*	*99.4*	*1.6*	***87.5***	***−7.16***	***0.00037***
	5	EL	72.5	19	62.5	−0.47	0.65
		*SM*	*83.3*	*11.8*	*75*	*−0.66*	*0.53*
	2	EL	53.3	7	58.3	0.66	0.54
		*SM*	*64.3*	*10.5*	***37.5***	***−2.39***	***0.053***

Results and statistical details for three different speeds. Bold indicates significantly lower (impaired) performance relative to controls (*t*-test results; Crawford and Howell, 1998): *t*(6) for SM, and *t*(4) for EL; SM’s data are italicized. Motion coherence thresholds of SM (right ventral lesion) were significantly impaired for the fast (27.27 °/s) and for the slow (5.4 °/s) motion, whereas those of EL, the left hemisphere patient, were normal, even when compared with younger control subjects (Fig. 1A).

#### Three dimensional structure-from-motion

**Figure 2:**
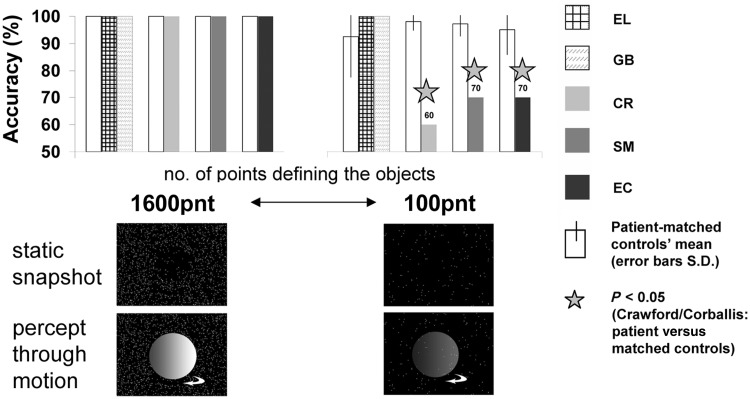
3D structure-from-motion performance. Display conventions as in Fig. 1. 3D structure-from-motion for many (*left*) and few points (*right*) defining the structure (see also Table 5). Below each histogram, a static snapshot from the motion display and an illustration of the percept caused by the motion are provided. All right ventral patients were impaired in 3D structure-from-motion when fewer points defined the structure, whereas left ventral patients performed at ceiling.

In this experiment, we assessed the patients’ ability to recognize 3D objects (spheres and cylinders) defined by motion cues (Fig. 2; Gilaie-Dotan *et al.*, 2011). The paradigm adopted allowed the presentation of an object (sphere or cylinder) based only on the local motion vectors across the object (Singer and Sheinberg, 2008). An animated 3D scene composed of a rotating object appearing in the centre of the screen and a static background was rendered in real-time as a pattern of points. A global percept of the moving object emerged from the integration of the local motion vectors into a coherent moving shape. As each point followed the trajectory of the underlying motion in the scene, only points located on the rotating object surface actually had local motion, while the points located ‘in the background’ did not. Each static frame of the animation appeared to be a uniform random field of points (Fig. 2, static snapshot). By varying the number of points in the display (and thus those defining the object), we were able to modulate task difficulty (see below).

Participants were first familiarized with the stimuli by viewing example trials with 1600 points and rotation speed of 0.5 rotations/s (corresponding to motion speed of 8.2–12°/s), parameters that provided easy recognition of the objects for all the patients (Fig. 2, 1600pnt condition), and reported verbally whether they saw a sphere or cylinder. After this, each condition included a session of 20 trials with fixed parameters (number of points, rotating speed). The participants’ task was to press a key once the object was recognized and then to report to the experimenter which object was present. The object rotated until the key was pressed without time restriction. Once the participant was ready, the next trial was initiated.

The experimental display was composed of flickering white points (diameter 0.16° visual angle) that randomly appeared on a black screen (‘formless dot field’). Points had a short lifetime (1.33 s, 80 frames at 60 frames/s) and their appearance was not synchronized. When a trial began, the motion of the rotating object was embedded into the flickering point display, so flickering points that appeared in the object’s location followed the local motion of the object’s surface for the full extent of their lifetime (1.33 s). When a trial ended, all the points in the display were stationary. In each run, half of the trials were of a rotating sphere and half of a cylinder (Fig. 2), and the order was determined randomly. The spinning object rotated around its north-south axis, which was tilted 27° away from the screen’s *y*-axis plane (north end farther away, south end closer), similar to the Earth’s tilt. The object rotation direction was determined randomly (clockwise or anticlockwise, 50% trials to each direction). The sphere and cylinder, viewed from 55 cm distance, subtended a visual angle of 12° × 9.9° (width × height) and 8.2° × 9.4° (width × height), respectively. Three conditions included a parametric change to the number of points composing the display (1600pnt, 500pnt, and 100pnt) while the rotation speed remained constant (0.5 rotations/s, Fig 2). Screen resolution was 1024 × 768, refresh rate 60 Hz. Stimuli were presented using MATLAB (Mathworks) and Psychophysics Toolbox 3 (Brainard, 1997). The experimental stimuli were based on the FDFDemo and moglFDF functions provided with the Psychophysics Toolbox 3, which provides an OpenGL (Silicon Graphics Inc.) interface for MATLAB.

Recognition accuracy was assessed for each participant for each condition. All patients and controls followed the same experimental order to rule out order-based learning effects. For each experimental condition, we determined whether performance was significantly different from that of the matched controls’ (Crawford and Howell, 1998; Crawford *et al.*, 2009), as presented in Table 5.

#### Motion detection

In this experiment, we assessed the ability to detect motion at different motion speeds. This was done after the 3D structure-from-motion experiment described above. The stimuli in this experiment were identical to those in the 3D structure-from-motion (see above) except that visual motion of dots (caused by the moving rotating object) was present in only half of the trials (10 of 20, randomly ordered within the session). In the remaining trials, there was no local visual motion.

Participants were familiarized with the stimuli and the instructions were given verbally before the experiment. On each trial, participants had to indicate verbally (‘yes’ or ‘no’) whether there was any motion in the centre of the screen. The experimenter terminated the trial immediately after the verbal response and then initiated the next trial. There was no time restriction for providing responses, and the motion persisted until the verbal response was given. There were two conditions: Fst of fast motion (0.5 rotations/s, average horizontal dot motion speed of 8.2–12°/s) of sparsely spaced points, and vSlw depicting very slow motion (0.0033 rotations/s, average horizontal dot motion speed of 0.055–0.08°/s) of densely spaced points. Motion detection accuracy was assessed for each participant for each condition, and we compared the patient’s accuracy to those of his/her corresponding control participants as described above (Crawford and Howell, 1998) (Table 4).

**Table 4:** Motion detection accuracy results and statistical analysis

Rotation/s	No. of points	Condition name		Accuracy (% correct)			T_Crawford_	P_Crawford_
			Patient initials	Controls (mean)	Controls (S)	Patient		
0.5	100	Fst	EL	100	0	100	0	1
			GB	100	0	100	0	1
			*CR*	*99.55*	*1.51*	***95***	***−2.89***	***0.016***
			*SM*	*99.44*	*1.67*	*100*	*0.32*	*0.76*
			*EC*	*98.33*	*3.54*	*100*	*0.45*	*0.67*
0.0033	1600	vSlw	EL	100	0	Normal*		
			GB	100	0	100	0	1
			*CR*	*100*	*0*	***80***	***−95742***	***4 × 10^−^^46^***
			*SM*	*99.44*	*1.67*	***50***	***−28.14***	***3 × 10^−^^9^***
			*EC*	*100*	*0*	***50***	***−94898***	***2 × 10^−^^37^***

Bold indicates that a patient’s detection accuracy was significantly impaired relative to their matching control subjects (*t*-test results; Crawford and Howell, 1998); right ventral lesioned patients’ data is italicized. ‘Fst’ condition depicts very rapid motion of sparsely spaced points (same presentation parameters as 100pnt condition in the 3D structure-from-motion experiment); ‘vSlw’ condition depicts very slow motion of densely spaced points. *EL’s detection performance for vSlw is missing but as she performed at 95% accuracy for object recognition in vSlw motion task (controls 68.75 ± 14.93), which detection is fundamental for, we are confident she is not impaired in detecting the motion at very slow speed (vSlw). In the easy Fst condition, all patients (but CR) were at ceiling (100% accuracy), 0% lapse rates (for CR 5% lapse rate). Note that for the very slow motion (vSlw) all right ventral lesioned patients were significantly impaired in detecting the motion (SM and EC at chance level) whereas the left hemisphere patients were normal (see also Fig. 1B). Furthermore, left hemisphere patients were also normal in very slow motion detection even when compared with younger control subjects, whereas right hemisphere patients were still impaired when compared with older control subjects (Supplementary material).

### Patients’ structural image acquisition

#### EL

EL’s anatomical magnetic resonance scans were acquired at the Brain Imaging Research Centre (BIRC) Pittsburgh on a Siemens Allegra MRI 3T scanner using a head coil, ∼1 year before her participation in this study and 14 years after her injury. The scan acquired 192 MPRAGE sagittal slices (1 mm thickness, inplane resolution of 1 × 1 mm^2^, matrix = 256 × 256, repetition time 1740 ms, echo time 3.04 ms, inversion time 1000 ms, flip angle = 8°).

#### GB

GB’s magnetic resonance clinical structural scans were acquired on a 1.5T GE Genesis Signa MR scanner equipped with a head coil, ∼3 years before her participation in this study. These included 23 axial T2 images (slice thickness = 5.5 mm, 7 mm gap, image size 512 × 512, pixel spacing 0.42968 × 0.42968 mm^2^, echo time = 96.512 ms, number of averages = 2, flip angle = 90°).

#### SM

SM’s MRI structural scans were acquired with identical parameters to those of EL’s (see above) at the Brain Imaging Research Centre (BIRC) Pittsburgh when he was aged 35. This was 17 years after his injury and ∼2 years before his participation in this study (for details, see Konen *et al.*, 2011).

#### CR

CR’s MRI structural scans were acquired at the Magnetic Resonance Research Centre, University of Pittsburgh Medical Centre on a 1.5T Signa whole body scanner (General Electric Medical Systems, Milwaukee, WI), ∼3 years after he had metabolic encephalopathy and ∼12 years before his participation in this study. This included 124 slices of 1.5 mm thickness with an in-plane resolution of 0.9375 × 0.9375 mm^2^, matrix of 256 × 256.

#### EC

EC’s CT clinical structural scans were acquired on a GE Medical Systems LightSpeed QX/i CT scanner when she was 40 years old, and ∼8 years before her participation in this study. These included 34 axial images without contrast with slice thickness of 2.5 mm (through the posterior fossa) and 7.5 mm (from the posterior fossa to the vertex), 512 × 512 image size, and pixel spacing of 0.449219 × 0.449219 mm^2^.

### Lesion delineation procedure

**Figure 3:**
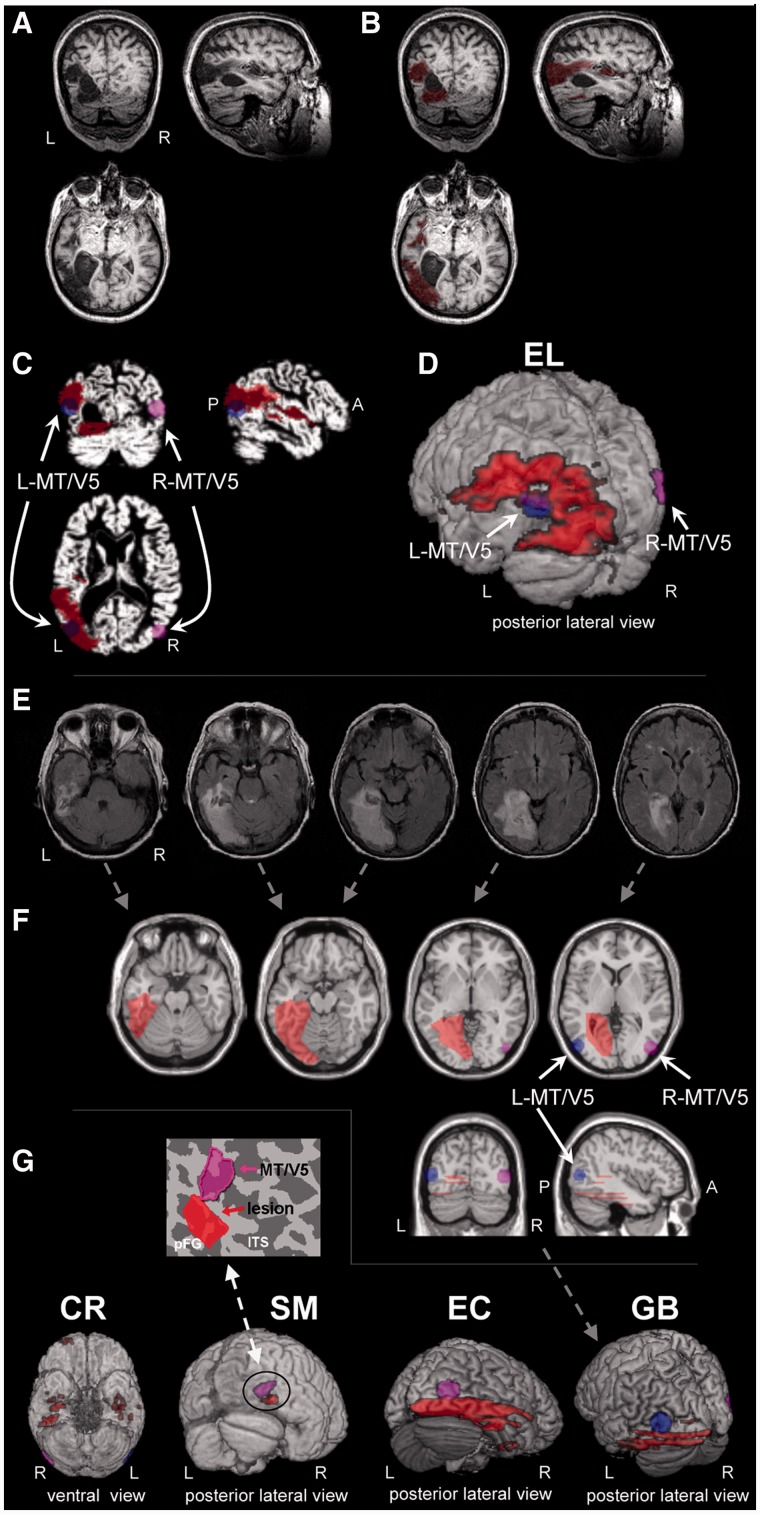
Lesion delineation procedure. Delineation for patients with high-resolution anatomical images (EL, SM and CR) followed the procedure depicted in **A–D**, whereas for patients with low spatial resolution images (GB and EC), the procedure is depicted in **E** and **F**. The original high resolution structural images of EL displayed in neurological conventions [(**A**), right on right] and the delineated lesion marked in red (**B**), were normalized into MNI, space (**C**; left/right MT/V5) is depicted in blue/pink. These are presented in a 3D overlay seen from a posterior left lateralized view (**D**). Patient GB’s original low resolution structural images (**E**) were used to approximate the lesion extent on a normalized MNI brain (**F**) as depicted in red on axial (*top*), coronal and sagittal views (*bottom*), along with the approximate location of L/R-MT/V5 as in **C**. (**G**) 3D lesion overlay of four of the patients (EL’s brain appears in **D**), viewed from posterior lateral views (CR viewed from ventral view). SM’s MT/V5 was functionally defined based on motion-sensitivity and retinotopy (unpublished observations), whereas for the other patients, MT/V5’s approximate localization (Kolster *et al.*, 2010) was used. Note that SM’s small lesion does not overlap his right MT/V5 (as also seen in the inset on flattened cortical map) and yet he shows significant motion perception impairments, whereas EL’s extensive lesion overlaps the expected location of left MT/V5 (**D**), yet her motion perception was normal.

For patients with high resolution anatomical images, the images were co-registered onto a T1 MNI canonical SPM image using SPM (http://www.fil.ion.ucl.ac.uk/spm), after which their lesions were traced manually in MRIcroN (http://www.cabiatl.com/mricro/mricro, see Supplementary material for tracing criteria) and saved as a binary image (Fig. 3A and B). For each patient, the co-registered anatomical images and the demarcated lesion were normalized into MNI space using the unified normalization segmentation of SPM (http://www.fil.ion.ucl.ac.uk/spm) as shown in Fig. 3C.

For GB and EC, who had low resolution anatomical images from the clinical scan, the lesion was manually traced onto the corresponding anatomical location in an MNI canonical SPM image (see Fig. 3E and F, and details in Supplementary material).

### Localization of motion sensitive regions

#### MT/V5

Using functional MRI, the location of SM’s motion-sensitive MT/V5 (Fig. 3G) was determined according to motion-sensitive activation in his medial temporal cortex (motion > static contrast at *P < *10^−25^, uncorrected), and this was further confirmed by retinotopy (unpublished observations). For the other patients, the locations of left MT/V5 and right MT/V5 were based on previously reported coordinates (Kolster *et al.*, 2010). Spheres with a 10 mm radius were created around these locations (Fig. 3C and F). The normalized brain and lesion, and the location of right and left MT/V5 were then loaded onto MRIcroN for display purposes for each patient (Fig. 3D and G). The location of the lesions was also compared with the assumed location of MT/V5 (Dumoulin *et al.*, 2000) to verify that the lesion did not invade the cortical sulci that were 1 cm away from the ITS-ALITS junction.

#### V3A

The location of V3A was based on previously reported coordinates (Tootell *et al.*, 1997*b*; Smith *et al.*, 1998; McKeefry *et al.*, 2010) and representative anatomical landmarks in its vicinity, such as the transverse occipital sulcus and its intersection with the intraparietal sulcus. For each patient, we examined the lesion in comparison with these coordinates and anatomical landmarks.

#### Parietal cortex

For each patient, we examined whether the lesion invaded parietal cortex in its more anterior and dorsal portion relative to V3A (see above), such as the intra-parietal sulcus or the superior parietal gyrus.

## Results

### Motion perception behavioural performance

To assess the contribution of the ventral stream to motion perception, we compared the performance of patients with that of controls on a series of tasks that have proven effective in uncovering impairments in motion perception (Vaina *et al.*, 1990; McLeod *et al.*, 1996; Spencer *et al.*, 2000; Milne *et al.*, 2002; Saygin, 2007; Gilaie-Dotan *et al.*, 2011).

#### Motion coherence

To characterize sensitivity to motion coherence, we calculated the proportion of coherently moving dots required to detect the fast coherent motion (27.27°/s) embedded in dots moving in random directions (Green, 1961; Levinson and Sekuler, 1976; Meteyard *et al.*, 2008; Gilaie-Dotan *et al.*, 2013).

The motion coherence thresholds for the two left ventral visual patients were normal (see details in Table 2 and Fig. 1A). In contrast, the motion coherence threshold for the patients with right ventral visual lesions was significantly impaired, with thresholds three to four times higher than those of the matched controls (see Table 2, Fig. 1A and Supplementary material for further supporting results and analyses). We were not able to obtain motion coherence thresholds from EC as she was no longer available for testing.

We further parametrically characterized motion coherence performance, in a wide range of motion speeds (5.4–27.27°/s) used in different studies. We measured EL’s (left lesion) and SM’s (right lesion) motion coherence discrimination accuracies (upwards versus downwards), while the proportion of coherent motion was modulated in a parametric fashion.

As detailed in Table 3 and displayed in Fig. 1A, and consistent with the motion coherence threshold results, EL, the left ventral patient, performed normally in all conditions, while the performance of SM, the right ventral patient, was out of the normal range for the slower (5.4°/s) and the fastest (27.27°/s) speeds, but not the medium speed (10.8°/s).

#### Motion detection

Further exploration of the patients’ basic non-form motion perception skills was done by having participants detect the motion of a coherently moving cluster of dots embedded in the centre of a random dot flickering field (Gilaie-Dotan *et al.*, 2011). The task was conducted under two conditions: in a fast motion condition (Fst, 8.2–12 °/s) in which a few fast-moving dots defined the motion, and in a slow motion condition (vSlw, 0.055–0.08°/s), when many slow-moving dots defined the motion. In the easy, fast-motion condition, when the motion cues are robust, all patients and their controls were at or close to ceiling (Table 4). In the more difficult, slow-motion task, while the left ventral patients were unimpaired (vSlw in Table 4), the patients with a right ventral visual lesion were all significantly impaired (Fig. 1B and Table 4). Note that the left ventral patients, whose accuracy was within normal limits, also displayed normal reaction times for motion detection (*P*’s > 0.8). In contrast, two of the right ventral patients (SM and EC), whose detection accuracies were not normal, were also significantly slower than their controls in their reaction times (EC: *P = *0.0012; SM: *P = *0.033).

#### Three dimensional structure-from-motion

The evidence above indicates that the right-lesioned individuals were impaired at basic non-object motion perception (except under very simple conditions as in the fast moving dots), but that the patients with left lesions performed normally. In this experiment, we assessed the patients’ recognition of 3D structures (sphere or cylinder) based on motion cues alone (termed structure-from-motion; Gilaie-Dotan *et al.*, 2011). Importantly, this experiment was done only with fast-moving dots defining the motion (8.2–12°/s, easily detectable by all patients, see above), whereas we parametrically manipulated only the number of dots defining the 3D structures. Figure 2 depicts the 3D structure-from-motion recognition results (Table 5). Recognition was at ceiling for all the patients and controls when many dots defined the form (1600pnt condition). The left ventral patients’ recognition was unimpaired (and actually at ceiling) even for the most difficult condition (100pnt), when only a few dots defined the motion. The right ventral patients’ recognition impairment, however, was revealed when less structural information was available in the display (i.e. fewer points defining the rotating structure, 500pnt or 100pnt). For the most difficult condition (100pnt), when only a few dots defined the motion, all the right ventral patients were significantly impaired in recognizing the rotating form (Fig. 2).

In sum, the left ventral patients performed within normal limits across the board in all motion perception tasks. In contrast, the right ventral patients displayed impaired 3D structure-from-motion, on top of their impairment in non-form motion perception.

**Table 5:** 3D structure-from-motion recognition accuracy and statistical analysis

Rotation/s	No. of points	Condition name		Accuracy (% correct)			T_Crawford_	P_Crawford_
			Patient initials	Controls (mean)	Controls (S.D.)	Patient		
0.5	1600	1600pnt	EL	100	0	100	0	1
			GB	100	0	100	0	1
			*CR*	*100*	*0*	*100*	*0*	*1*
			*SM*	*100*	*0*	*100*	*0*	*1*
			*EC*	*100*	*0*	*100*	*0*	*1*
	500	500pnt	EL	100	0	**95**	**−5000**	2*10^-11^
			GB	100	0	100	0	1
			*CR*	*100*	*0*	*100*	*0*	*1*
			*SM*	*100*	*0*	***90***	***−9486***	2*10^-29^
			*EC*	*100*	*0*	*100*	*0*	*1*
	100	100pnt	EL	92.50	15	100	0.5	0.651
			GB	92.50	15	100	0.5	0.651
			*CR*	*98.18*	*3.37*	***60***	***−10.84***	8*10^-7^
			*SM*	*97.22*	*4.41*	***70***	***−5.857***	4*10^-4^
			*EC*	*95.00*	*9.01*	***70***	***−2.631***	***0.03***

Display conventions as in Table 4. In the easy 1600pnt condition, all patients and all controls were at ceiling (100% accuracy, 0% lapse rates). EL’s recognition at 500pnt was likely due to a verbal mistake, because at 100pnt, which is much more difficult, she was at ceiling. When less form information was provided (100pnt) all right ventral lesioned patients were significantly impaired, whereas the left ventral lesioned patients remained at ceiling performance (see also Fig. 2). Left hemisphere patients were in the normal range even when compared with younger control subjects, while right hemisphere patients were still impaired when compared with older controls (Supplementary material).

### Lesion comparisons

The behavioural findings implicate the right, but not the left ventral visual pathway, in contributing to normal central motion perception at a wide-range of motion speeds (detection at speed of 0.055–0.08°/s, coherence at speeds of 5.4 and 27.27°/s) and this was true even for tasks that did not involve form processing (motion coherence and motion detection). One potential, simple explanation for this result might be that in patients with right but not left hemisphere damage, the lesion impinged on the motion sensitive region MT/V5, or on the relatively motion sensitive V3A. To rule out this possibility, we delineated each patient’s lesion based on previously acquired anatomical images (Fig. 3) and situated the lesion relative to the expected location of MT/V5 and of V3A. Lesion delineation was performed in native space and the images were then transformed into normalized Montreal Neurological Institute (MNI) space for comparison with the location of the middle temporal motion-sensitive region MT/V5 as defined functionally (for SM), or, for the other patients, as reported in the literature (Watson *et al.*, 1993; Dumoulin *et al.*, 2000; Kolster *et al.*, 2010). Lesion location was also compared with the V3A location as reported above (see ‘Materials and methods’ section), and with parietal cortex. Table 6 provides a summary of the lesion volume, and determination of whether or not each lesion overlapped MT/V5, V3A, or parietal cortex. Although for both GB (left lesion) and EC (right lesion) we cannot definitively conclude whether MT/V5 is spared due to the relatively low spatial coverage of their clinical structural images, for GB it is highly likely that her left MT/V5 is spared based on Dumoulin *et al.* (2000). With respect to V3A, we can conclude with high probability that their lesions did not overlap right or left V3A. However, we urge caution in interpreting these two cases.

**Table 6:** Summary of patients’ lesions, and performance on motion perception tasks

	Left ventral lesion		Right (+ left ?)	Right ventral lesion	
	Patient EL	Patient GB	Patient *CR*	Patient *SM*	Patient *EC*
Lesion extent	Extensive	Extensive	Intermediate	Small	Extensive
Lesion approximate size iso space (mm^3^)	43028	?	1510	952	?
Lesion conservative size iso space (mm^3^)	29616	?		516	?
MT/V5 overlap	Yes	?	No	No	?
V3A overlap	?	No	No	No	No
Parietal cortex overlap	No	No	?	No	No
**Motion perception tasks**					
Motion coherence threshold	Normal	Normal	*Impaired*	*Impaired*	*Unknown*
Motion coherence accuracy	Normal	Unknown	*Unknown*	*Impaired*	*Unknown*
Motion detection (very slow)	Normal	Normal	*Impaired*	*Impaired*	*Impaired*
Structure-from-motion (3D)	Normal	Normal	*Impaired*	*Impaired*	*Impaired*

The approximate lesion size was based on the lesion delineating procedure, and an additional conservative size estimate is also provided (see ‘Materials and methods’ section). Assessment of lesion overlapping MT/V5 is based on functional localization for SM, and on MT/V5 reported location for the other patients (Dumoulin *et al.*, 2000; Kolster *et al.*, 2010), for V3A and parietal overlap and more details see ‘Materials and methods’ section. Due to the low spatial coverage of GB and EC’s clinical scans, we were not able to estimate the lesion size or conclusively determine MT/V5 lesion overlap. Motion perception classifications (*normal or impaired*) for each patient is with respect to their control group.

In the individuals with left ventral damage (EL and GB), the lesions were extensive, whereas in the patients with right ventral damage, the lesions varied both in size (SM small, EC extensive, and CR scattered) and location. Importantly, however, neither the size nor location of the lesion with respect to MT/V5 or V3A were correlated with the impairment in motion perception: as evident from Table 6, despite the extensive left ventral lesion, likely overlapping left MT/V5 and perhaps her left V3A, EL’s motion perception was normal on all tasks, from basic motion perception to more complex structure-from-motion tasks. GB’s motion perception was also normal on all tasks despite an extensive left ventral lesion. In contrast, SM, who has a small lesion in the right ventral cortex with clear sparing of right MT/V5 (and normal activation of MT/V5 as revealed through functional neuroimaging; unpublished observations), clear sparing of his right and left V3A and his parietal cortex, and clear sparing of fibre connectivity in his posterior brain regions (revealed by a diffusion tensor imaging study; Jung and Jung, 2010), was significantly impaired on all motion perception tasks apart from motion coherence at medium speed level (10.8 °/s). CR, who has a right ventral lesion along with some other punctate abnormalities, but spared right and left MT/V5 (further confirmed by a neuroradiologist blind to the purpose of study) and spared V3A, was also significantly impaired in all motion perception tasks. EC, who has an extensive right ventral lesion, with a clear sparing of her V3A and parietal cortex, was impaired in all motion perception tasks tested.

The dissociation between behaviour and the presence of a MT/V5 lesion is apparent: from EL and from GB, we can conclude that central motion perception can be normal even with a lesion to left MT/V5, and from SM and CR, we can conclude that despite spared right and left MT/V5 (for EC clear sparing of left MT/V5), central motion perception can be impaired at all levels and for very fast and slow motions (except for intermediate speed of 10.8°/s). We can also rule out lesion size as being a factor in the perceptual impairment: EL and GB have extensive lesions yet spared central motion perception, whereas SM and CR have small or intermediate size lesions but are impaired at even very basic central motion perception. Taken together, these data suggest that these motion perception impairments are independent of MT/V5 integrity, are not attributable to damage to V3A or parietal cortex, and might not be correlated with lesion size (*cf*. Saygin, 2007).

## Discussion

The goal of this study was to explore the functional contribution of the ventral visual cortex to the ability to perceive motion. Five patients with a lesion to either the right or left ventral visual cortex, participated in a range of psychophysical tasks examining the perception of both non-form-based motion and form-based motion (e.g. structure-from-motion), all centrally displayed, at a wide range of motion speeds. Two major results were obtained. First, surprisingly, in addition to the impairment in perceiving form-based motion, even basic motion perception (such as detecting the presence of motion in a display or discerning the coherence of random moving dots) was adversely affected by ventral visual pathway lesions for slow as well as for fast motion displays. Second, the perturbation in the perception of motion was only observed in those patients with right ventral lesions, whereas the motion perception of those with left ventral lesions remained in the normal range for all tasks.

Our novel findings suggest that the perception of motion in the central part of the visual field, even when the motion is not in the context of form or structure processing, is dependent on the integrity of the right ventral visual cortex. This dramatic finding indicates that motion-sensitive areas of cortex by themselves do not suffice for normal motion perception and that additional cortical regions are required to support motion perception.

### Ventral visual cortex affects motion perception

Here, we have demonstrated, for the first time, that ventral stream integrity is necessary for uncompromised motion perception, and this is true even for the detection of basic motion information. The finding that even basic, non-form motion perception relies on (right) ventral stream integrity runs counter to prevalent views in neuroscience. Motion inputs are often assumed to be processed in specialized, perhaps even dedicated, motion-sensitive cortical regions such as MT/V5, MST (Zeki, 1974; Newsome and Pare, 1988; Tootell *et al.*, 1995; Heeger *et al.*, 1999; Rees *et al.*, 2000; Kolster *et al.*, 2010) and V3A (Tootell *et al.*, 1997*b*; McKeefry *et al.*, 2010), and the activity of these regions is functionally correlated with motion perception (Newsome *et al.*, 1989; Salzman *et al.*, 1992; Celebrini and Newsome, 1994; Zohary *et al.*, 1994; Bradley *et al.*, 1998; Heeger *et al.*, 1999; Grunewald *et al.*, 2002; McKeefry *et al.*, 2010; Takemura *et al.*, 2012). However, recent studies indicate that MT/V5 responses depend on local rather than global motion, and suggest that motion perception relies on activity in multiple brain regions, not just on MT/V5 (Majaj *et al.*, 2007; Hedges *et al.*, 2011). Some of these additional regions are assumed to be associated with the dorsal visual stream, which is not too surprising given that the experimental paradigms adopted typically tap motion processing in the context of action planning, saccadic movements or attention, all preferentially engage the dorsal pathway (Newsome *et al.*, 1989; Treue and Maunsell, 1996; Newsome, 1997). Although ventral stream regions have also been implicated in motion processing (De Valois *et al.*, 2000; Tolias *et al.*, 2005; Lu *et al.*, 2010; Li *et al.*, 2013), and there have been suggestions that intact motion perception relies on a combination of dorsal and ventral-related contributions (De Valois *et al.*, 2000), and that ventral cortex might be important for slow motion while dorsal cortex is important for fast motion (Gegenfurtner and Hawken, 1996; Thompson *et al.*, 2006; Hayward *et al.*, 2011), the necessary role of ventral visual cortex in motion perception has not been demonstrated. Our findings implicate ventral visual cortex in central motion perception; this is true, critically, not only for slow motion (at 0.055–0.08 °/s, as in the motion detection task, or 5.4°/s in the motion coherence task), but also for very fast motions (motion coherence at 27.27°/s). Moreover, we have argued, that the ventral contribution is not limited to contexts in which form representations are required or evoked. Although the ventral visual cortex has not standardly been considered a critical substrate for motion perception, and, instead, has been considered a downstream (feed-forward) recipient of motion signals that are relevant for shape perception (Sary *et al.*, 1993; Grill-Spector *et al.*, 1998; Kriegeskorte *et al.*, 2003; Peuskens *et al.*, 2004), there is some evidence consistent with our findings. First, ventral visual areas do exhibit motion sensitivity. Electrophysiology in macaque ventral cortex has revealed that there is a direct motion selective pathway from V1 to V2 that bypasses MT/V5 (Gur and Snodderly, 2007), that 10–30% of V4 neurons are direction selective (Desimone and Schein, 1987; Mountcastle *et al.*, 1987; Ferrera *et al.*, 1994), and that V4 is sensitive to changes in motion direction (Tolias *et al.*, 2005). Furthermore, optical imaging has shown that macaque V2 and also V4 contain a columnar organization of motion directional maps in the foveal aspects of its representation (5° visual angle; Lu *et al.*, 2010; Li *et al.*, 2013), in line with our central displays in this study. Second, direct bilateral connectivity and interdependency exists between ventral stream and lateral temporal motion sensitive regions (e.g. between MT/V5 and V2, V4; Maunsell and Van Essen, 1983; Ungerleider and Desimone, 1986), and a relatively new motion sensitive region, discovered in the ventral aspects of macaque superior temporal sulcus, suggests an additional motion-sensitive processing route (Nelissen *et al.*, 2006). Finally, area MT/V5, the pre-eminent motion area, shows sensitivity to ‘static’ object shape in the presence and even in the absence of implied motion cues (Kourtzi and Kanwisher, 2000; Kourtzi *et al.*, 2002).

An alternative and possibly simpler explanation of our results, however, is that the impairment in motion perception following a right ventral lesion might result from compromised white matter tracts providing inputs to MT/V5, or from lesioned V3A or parietal cortex, rather than from the ventral lesion itself. This is unlikely to be the case here. First, the location of the lesion in the three patients with right ventral damage (and impaired motion perception) differed along the ventral stream; whereas SM’s lesion was close to but distinct from his right MT/V5, CR’s lesion was far more anterior and not in the vicinity of MT/V5 or V3A. Second, according to our lesion delineation, but also as revealed by functional MRI data, SM evinces a normal pattern of motion-selective activity in the standard MT/V5 regions (unpublished observations). Third, as revealed by diffusion tensor imaging, SM’s white matter tracts in posterior brain regions were intact (Jung and Jung, 2010), so it is unlikely that disrupted connectivity to/from his MT/V5 or V3A was the source of his motion perception impairments. For CR and EC, although we cannot rule out this scenario completely, it seems unlikely that their MT/V5 white matter connectivity is damaged following an examination of their anatomical images. EC’s lesion is restricted to the ventral aspect of the occipital cortex, i.e. medially, more ventral to the calcarine sulcus, and laterally, more ventral to the lateral occipital sulcus, and CR’s lesion does not seem to invade the occipital retinotopic regions. Fourth, the lesions of the right ventral patients were restricted to ventral cortex and did not impinge on MT/V5, V3A or parietal cortex. Taken together, these results indicate that the ventral stream is not just a recipient of motion inputs from motion sensitive regions, but is likely an active and critical player in supporting motion perception.

How can we then reconcile the critical role of the ventral visual stream in motion perception, as shown here, with the previously reported critical role of MT/V5 (Newsome and Pare, 1988)? Both sides of this equation require further explication, especially when EL, whose lesion affected left MT/V5, was not impaired in motion perception, whereas SM, with undamaged bilateral MT/V5 was impaired in motion perception. We suggest two possible resolutions to this paradox. The first is based on the idea that intact motion perception depends on both normal central and peripheral motion perception. We speculate that while motion-sensitive visual regions such as MT/V5, MST and dorsal stream are critical for peripheral normal motion perception covering the whole visual field, ventral visual cortex is critical for central motion perception (Hasson *et al.*, 2002; Malach *et al.*, 2002). According to this division of labour, lesions to right ventral cortex would give rise to central field motion perception impairments, whereas lesions to MT/V5 would cause contralateral visual field motion perception impairments, perhaps sparing central motion perception. SM, with intact right and left MT/V5, is impaired in central motion perception following his right ventral visual lesion. EL’s central visual motion perception is normal despite her lesioned left MT/V5, presumably due to her undamaged right ventral visual cortex, and to overlapping receptive fields for central motion by bilateral MT/V5. Previous data are in line with this resolution, as half-field akinetopsia due to contra lateral MT/V5 lesion can be attributed to impaired peripheral motion perception as impairments are more evident with off-centre stimuli (Newsome and Pare, 1988). Indeed, akinetopsic patient LM, is reported to show bilateral motion blindness that is more apparent in the periphery; her movement perception is somewhat preserved up to 15° of eccentricity, whereas for peripheral visual field her motion sensitivity is limited to discriminating moving and stationary targets (Zihl *et al.*, 1983). A hypothetical scenario is one in which the dorsal stream detects an object moving across the visual field. Once the eyes are moved and the object occupies foveal vision, ventral stream motion processing becomes critical. An alternative second possible resolution suggests that while MT/V5’s role is processing the basic building blocks of motion stimuli, right ventral cortex spatially integrates basic motion cues into holistic moving percepts such as surfaces or objects. Both of these suggested resolutions independently lead to a possible prediction based on the action-perception division of labour within the visual system (Goodale and Milner, 1992), that although dorsal stream function is critical to processing visual motion leading to action, the ventral stream is critical for processing visual motion leading to perception. If this holds true then it might support independent motion processing in dorsal and ventral streams, each dedicated to their different purpose. Further evidence and testing will be necessary to accept or refute these theoretical alternatives.

### Differential hemispheric contributions to motion perception

In addition to uncovering a critical role for ventral cortex in motion perception, our findings suggest a hemispheric asymmetry in this process, as impairments were evident only following a lesion to the right hemisphere. We speculate that bilateral human motion-sensitive regions such as MT/V5 and MST (Tootell *et al.*, 1995; Heeger *et al.*, 1999; Rees *et al.*, 2000; Kolster *et al.*, 2010) and also V3A (Tootell *et al.*, 1997*a*; McKeefry *et al.*, 2010) may well process motion equivalently, and that the laterality effect we have observed is related to the dominance of the right hemisphere in form and object perception. Although form and object representations activate both hemispheres (Gilaie-Dotan *et al.*, 2008, 2010), neuropsychological studies suggest that the right ventral cortex plays a more critical role in object recognition (Humphreys and Riddoch, 1984; Davidoff and Warrington, 1999; Barton, 2011; Konen *et al.*, 2011). Left ventral lesions do not typically give rise to profound agnosia, and instead, tend to result in deficits in visual word recognition (Behrmann *et al.*, 1998; McKeeff and Behrmann, 2004). This asymmetry is supported in our patient sample, with the right ventral patients more profoundly impaired in object recognition than the left ventral patients (Behrmann *et al.*, 2006). Since visual motion usually occurs when an object with a particular form is moving, we speculate that motion processing is tightly linked to form processing (Kourtzi *et al.*, 2008) even for non-form motion, and therefore, the right ventral visual dominance for form perception might serve as the basis of the motion perception laterality effect we have observed in this study. Needless to say, these speculative assertions will need to be confirmed in future studies.

## Conclusion

Our study reveals that the ability to perceive slow and fast moving stimuli appearing in central vision is dependent on ventral visual cortex integrity, especially in the right hemisphere. This finding suggests a tight interplay between ventral visual cortex and motion perception, that has not been observed to date, and, hence, challenges the received view that a network of brain areas in the dorsal stream suffices in supporting normal motion perception. Instead, these results license an account in which normal motion perception is subserved by a more distributed functional circuit, including ventral visual cortex.
